# *Centaurea lycaonica* Extracts Induce Apoptosis in HeLa Human Cervical Cancer Cells via Bax/Bcl‐2 Modulation and Caspase Activation: An LC‐HRMS‐Based Study

**DOI:** 10.1002/fsn3.70528

**Published:** 2025-07-15

**Authors:** Ayşe Kübra Karaboğa Arslan, Safa Eminoğlu, Leyla Paşayeva, Nuh Mehmet Bozkurt, Osman Tugay

**Affiliations:** ^1^ Department of Pharmacology, Faculty of Pharmacy Erciyes University Kayseri Türkiye; ^2^ Department of Pharmacognosy, Faculty of Pharmacy Erciyes University Kayseri Türkiye; ^3^ Department of Pharmaceutical Botany, Faculty of Pharmacy Selçuk University Konya Türkiye

**Keywords:** Bax, Caspase‐3, *Centaurea lycaonica*, HeLa

## Abstract

The *Centaurea* species (Asteraceae) is widely used in folk medicine. However, its apoptotic effects have not been adequately determined. *Centaurea lycaonica* is an endemic species belonging to the *Centaurea* L. genus. In this study, methanol (CHM) and dichloromethane (CHD) extracts obtained from the aerial part of *C. lycaonica* were treated to human cervical carcinoma (HeLa) cells to investigate their cytotoxic effects using Sulforhodamine B (SRB) assay and xCELLigence Real‐Time Cell analyzing system, in addition to their apoptotic effects by measuring caspases. As a result, the IC_50_ value of CHD and CHM on HeLa cells was calculated respectively as 39.27 μg/mL and 516.50 μg/mL. Increased activity of caspase‐3 and ‐9, along with a higher Bax/Bcl‐2 ratio, was observed in the HeLa cells at 30 and 100 μg/mL concentrations for CHD and 240 μg/mL for CHM. Significant cytotoxic effects through the mitochondrial apoptotic pathway were shown for the HeLa cells. LC‐HRMS determined the phytochemical content of extracts. The major compounds, diosmetin, and apigenin, were detected in the CHD extract, and chlorogenic and quinic acid were detected in the CHM extract. Apoptosis is induced primarily by CHD, which contains a high amount of diosmetin and is a promising candidate for anticancer research.

## Graphical abstract

This study highlights the pharmacological mechanisms of dichloromethane (CHD) and methanol (CHM) extracts of *Centaurea lycaonica* Boiss. & Heldr. herba as the induction of apoptosis on HeLa cells. Apoptotic properties of CHD and CHM are through suppression of Bcl‐2 and increase in Bax, caspase 9, and 3 activities on HeLa cells. The dichloromethane extract increases the cytotoxic activity of HeLa cells. The major compounds in active CHD extract were detected as diosmetin.

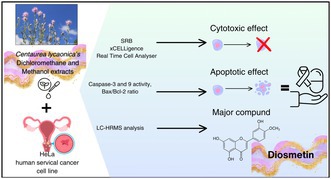

**Abbreviations**
BCA: Bicinchoninic acid
CHD: Dichloromethane exrtact of *Centaurea lycaonica*
CHM: Methanol extract of *Centaurea lycaonica*
Dox: Doxorubicin
HeLa: Human cervical carcinoma‐derived
HPV: Human papillomavirus
IC_50_
: Half‐maximal inhibitory concentration
RTCA: Real‐Time Cell analyzing

## 1 Introduction

Among the 450–500 species of *Centaurea*, there is limited research on the toxicity of *Centaurea* species (Çelikezen et al. 2019; Carev et al. 2022; Escher et al. 2018; Radan et al. 2017; Hadjira et al. 2021). Since the current chemotherapy drugs are associated with numerous acute and chronic serious side effects, many studies aim to find new drugs with as few side effects as possible (Seelinger et al. 2012). Anticancer drugs derived from natural sources such as plants, microorganisms, and marine organisms are the most widely clinically used drugs (> 60%) in the last century due to their high efficiency and low side effects (Seelinger et al. 2012).

The *Centaurea* genus, a member of the Asteraceae family, is widely distributed. Some species of *Centaurea* have been used in traditional medicine to treat malaria, colds, diarrhea, and ulcers (Arif et al. 2004). The endemic Turkish *Centaurea lycaonica* Boiss. & Heldr., locally known as “zarif düğme”, is a plant species belonging to the Asteraceae (Compositae) family and the *Centaurea* genus. The plant's distribution is exclusive to the western and central Anatolia region in Türkiye. It was first collected near Konya city by Heldreich in 1845. Due to the lack of detailed information about its distribution, scientists could not locate it for a long time. Recently, the *C. lycaonica* species was found, collected, and identified again near Konya city (Uysal et al. 2008).

**FIGURE 1:**
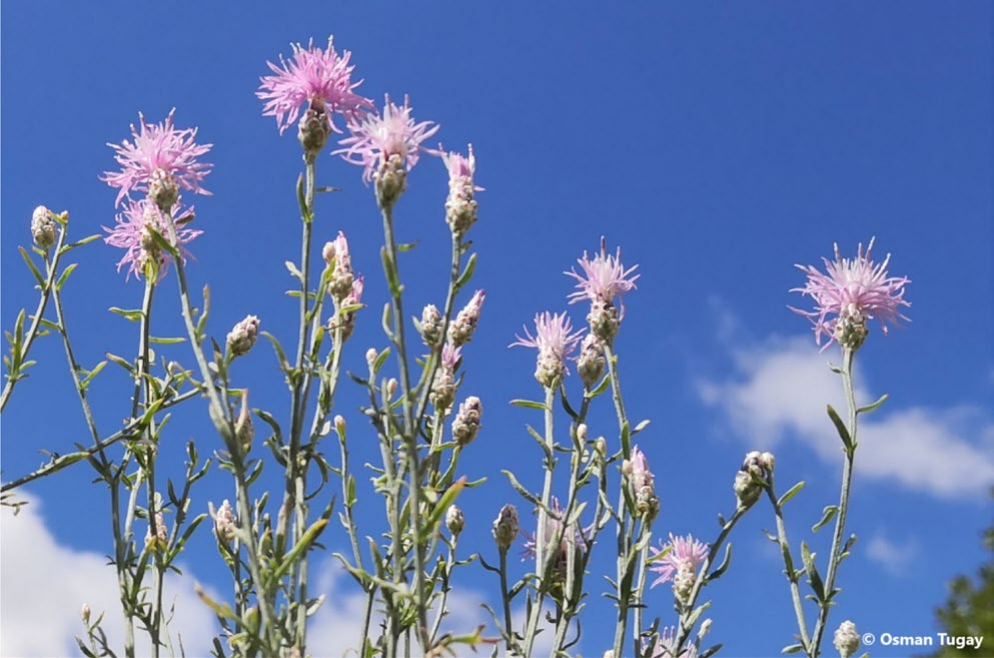
*Centaurea lycaonica* Boiss. & Heldr. (Photo: Prof. Dr. Osman Tugay).

*C. lycaonica* is a perennial plant in meadows, pastures, and dry slopes. Like many *Centaurea* species, the plant's flowers are blue‐purple and consist of small flower heads (Figure 1). The leaves are generally hairless with serrated edges. Research on *C. lycaonica* is limited, but some studies have been conducted on its biology, ecology, and medicinal potential (Özel 2008). The plant's antimicrobial, antioxidant, and enzyme inhibition activities have also been investigated (Fatullayev et al. 2023). The effects of salt stress on the biochemical and physiological properties of *C. lycaonica* have also been studied (Yıldıztugay et al. 2011). *Centaurea* species are promising candidates for developing a natural plant source for anticancer agents due to its content of various bioactive compounds that induce apoptosis and suppress the proliferation of cancer cells (Çelikezen et al. 2019; Carev et al. 2022; Escher et al. 2018; Radan et al. 2017; Hadjira et al. 2021). Numerous studies have demonstrated that certain species of *Centaurea* contain diosmetin (3′,5,7‐trihydroxy‐4′‐methoxyflavone) (Bozkurt 1997; Tüfekçi et al. 2024; Milošević Ifantis et al. 2013; Ercan et al. 2025). A bioflavonoid compound known to trigger apoptosis by inducing G2/M phase cell cycle arrest and activating the mitochondria‐mediated intrinsic apoptotic pathway. This includes downregulation of antiapoptotic proteins such as Bcl‐2 and Bcl‐xL, along with upregulation of proapoptotic proteins like cleaved PARP, Bax, and cleaved caspase‐3 (Pan et al. 2023). Considering the lack of cytotoxic and phytochemical studies on *C. lycaonica* in the existing literature, this article aims to contribute to exploring a natural plant source with potential for medicinal development.

This study aimed to investigate the cytotoxic effects of dichloromethane (CHD) and methanol (CHM) extracts from the endemic *C. lycaonica* species on HeLa human cervical cancer cells, with a focus on elucidating the apoptotic mechanisms through the assessment of caspase‐3 and caspase‐9 activities, as well as the Bax/Bcl‐2 ratio, and to identify the bioactive compounds responsible for these effects using LC‐HRMS analysis.

## 2 Materials and Method

The dichloromethane (CHD) and methanol (CHM) extracts of the endemic *C. lycaonica* species were obtained. The cytotoxic effects of these extracts on the human cervical cancer cell line (HeLa) were determined using Sulforhodamine B (SRB) viability assay and xCELLigence Real‐Time Cell Analysis (RTCA) system. Subsequently, apoptotic cell death was investigated by measuring the activity of caspase‐3, caspase‐9, and the Bax/Bcl‐2 ratio. However, LC‐HRMS analyses were carried out to detect activity‐responsible compounds.

### 2.1 Plant Material

*C. lycaonica* species were collected from the steppe of Seydişehir (37°42′40″ N, 32°04′18″ E) in the area of Konya province in Türkiye in August by Prof. Dr. Osman TUGAY was given a herbarium number (KNYA Herb. No: 30.214). The aerial parts of the species dried in a shaded, well‐ventilated environment.

### 2.2 Preparation of Plant Extract

For this study, a dichloromethane extract was prepared using the maceration method for 24 h. Subsequently, the extracted plant material was extracted with methanol (Paşayeva et al. 2025). Then, the extracts were evaporated to dryness under low pressure at 38°C in a rotavapor. The obtained extracts were lyophilized and stored at −20°C until use.

### 2.3 HeLa Cell Line and Culture

HeLa cell line was obtained from the Department of Pharmacology at Erciyes University/Türkiye. The medium used for the proliferation of HeLa cells was prepared with DMEM supplemented with 10% FBS (Biochrom S0115) and 1% penicillin/streptomycin (Capricorn CP 18‐2207). HeLa cells were regularly passaged and incubated (İncu safe MCO‐18AIC) at 37°C in a standard cell culture atmosphere containing 5% CO_2_ and 95% humidified air. The prepared extracts were dissolved in DMSO, and the final DMSO concentration in the medium was less than 0.1%. The extracts were prepared with fetal bovine serum‐free (FBS‐free) DMEM (Dulbecco's modified Eagle's medium) (Sartorius 230901812) to be used in the cytotoxicity and apoptosis investigations.

The cytotoxic effect of *C. lycaonica* extracts that CHD and CHM on cell viability was first assessed using the SRB assay for a general concentration screening. Subsequently, specific concentrations were determined from the initial screening and evaluated using the SRB assay and the xCELLigence RTCA Single Plate (SP) system (ACEA Biosciences).

### 2.4 SRB Viability Assay

The assay was applied as described by Vichai and Kirtikara (2006). HeLa cells were seeded in 96‐well plates at a density of 1 × 10^4^ per well and allowed to adhere for 48 h in a 37°C incubator with 5% CO_2_. The cells were then treated with CHD concentrations (10, 15, 20, 30, and 100 μg/mL) and CHM (10, 30, 100, 180, and 200 μg/mL) extracts prepared in FBS‐free DMEM and control wells. The positive control wells were treated with doxorubicin (Dox) (0.3, 0.5, 0.75, 1 and 1.5 μM). The wells were incubated for 48 h and were fixed by adding 10% cold trichloroacetic acid (TCA) (Sigma T6399‐100 g) to each well and incubating at 4°C. At room temperature, the plates were washed several times, air‐dried, and stained with SRB solution in 1% acetic acid (Sigma 64‐19‐7). Excess dye was removed. The protein‐bound dye was solubilized in 10 mM Tris base solution, and absorbance was measured at 510 nm using a microplate reader (Biotek Synergy HT). The absorbance readings were used to calculate the percentage of cell viability.

### 2.5 xCELLigence RTCA System

HeLa cells were seeded into E‐plates (Roche 05232368001) at an appropriate density and allowed to adhere for 24 h in a 37°C incubator with 5% CO_2_. The cells were then treated with CHD concentrations (10, 15, 20, 30, and 100 μg/mL) and CHM (10, 30, 100, 180, and 200 μg/mL) extracts prepared in FBS‐free DMEM and control wells. The positive control wells were treated with Dox (1 and 1.5 μM). The E‐plates were placed into the xCELLigence RTCA SP system, and the impedance was measured every 15 min for 72 h. The impedance measurements were converted to cell index (CI) values, which reflect the cells' number, viability, and morphology.

### 2.6 Apoptotic Analysis

The activity of caspase‐3, caspase‐9, Bax, and Bcl‐2 was assayed according to the manufacturer's instructions (SunRed Biotechnology Co. Ltd., Shangai, PCR). HeLa cells were seeded into 6‐well plates at a density of 1 × 10^6^ cells per well and allowed to adhere for 24 h in a 37°C incubator. The cells were then treated with various concentrations of CHD (30 and 100 μg/mL) and CHM (240 μg/mL) extracts and Dox (1 μM) prepared in FBS‐free DMEM and were incubated for 48 h. The culture medium was removed, and the cells were washed with cold phosphate‐buffered saline (PBS) (AppliChem A9177‐100 tablet). Cold lysis buffer containing protease inhibitor (A.G. Scientific B1352) was added to the cells and kept on ice for 30 min. The cell lysate was collected by scraping the cells off the plate, centrifuged at 1000 rpm for 10 min, at 4°C, and sonicated using an ultrasonicator (Bandelin Sonopuls Hd 2070). Protein content was measured by using the bicinchoninic acid (BCA) protein assay kit (Cell Signaling 7780).

### 2.7 LC‐HRMS Analysis

The bioactive substances in the active extract were determined by full scan high‐resolution accurate mass spectrometry (LC–HRMS). LC‐HRMS analyses were performed using a DIONEX UltiMate 3000 RS pump, DIONEX UltiMate 3000 RS autosampler, and a DIONEX UltiMate 3000 RS column oven‐equipped LC system and Exactive Plus Orbitrap (ThermoFisherScientific) high‐resolution MS with heated electrospray ionization interface. The Orbitrap HRMS, equipped with a heated electrospray ionization interface, was operated in both positive (Full MS/AIF) and negative (Full MS/AIF) modes. For separation the column Phenomenex Gemini 3 μm NX‐C18 110 Å (100 mm × 2 mm) was used and column temperature was 30°C. The mobile phase was selected as 0.5% (v/v) acetic acid (A) and methanol (B) (v/v) and flow rate 0.3 mL/min. The crude extract was subjected to filtration through a 0.22 μm PTFE syringe filter before injection to remove any particulate matter. The filtered sample was diluted to a final concentration of 1 mg/mL in 50% methanol and injected in triplicate for quantitative analysis. In this analyses, the gradient elution was used, starting at 0% B and then increasing to 98% B in 13.0 min, holding at 98% B for 2.0 min and then lowering back to 0% B in 16.0 min. The total run time was 20.0 min (Kho et al. 2015).

For each identified phytochemical, a series of standard solutions were prepared at the following concentrations: 10, 20, 40, 60, 80, 100, 200, 400, 600, 800, and 1000 μg/mL. Each standard was injected in triplicate. Calibration curves were generated for each compound by plotting the peak area versus concentration, and all curves showed linearity (*R* ^2^ > 0.995). Quantification of metabolites in the extract was carried out by referencing the calibration curves derived from the standard compounds. Each metabolite concentration was calculated based on its corresponding linear regression equation.

### 2.8 Statistical Analysis

All calculations from xCELLigence were obtained using the RTCA‐integrated software of the xCELLigence system. Statistical analysis was performed by GraphPad Prism Software Version 8.3.0 (La Jolla, CA, USA) to compare differences in values between the control and experimental groups for SRB and apoptotic assay analysis. The results are expressed as the mean ± SD calculated from 3 separate experiments. Statistically significant values were compared using one‐way ANOVA and Dunnett's post hoc test, and statistical significance was determined by *p* values < 0.05. Significance intervals were determined as *< 0.05; **< 0.01 and ***< 0.001.

## 3 Results

### 3.1 Effects of the *C. lycaonica* Extracts on the Cell Viability

**FIGURE 2:**
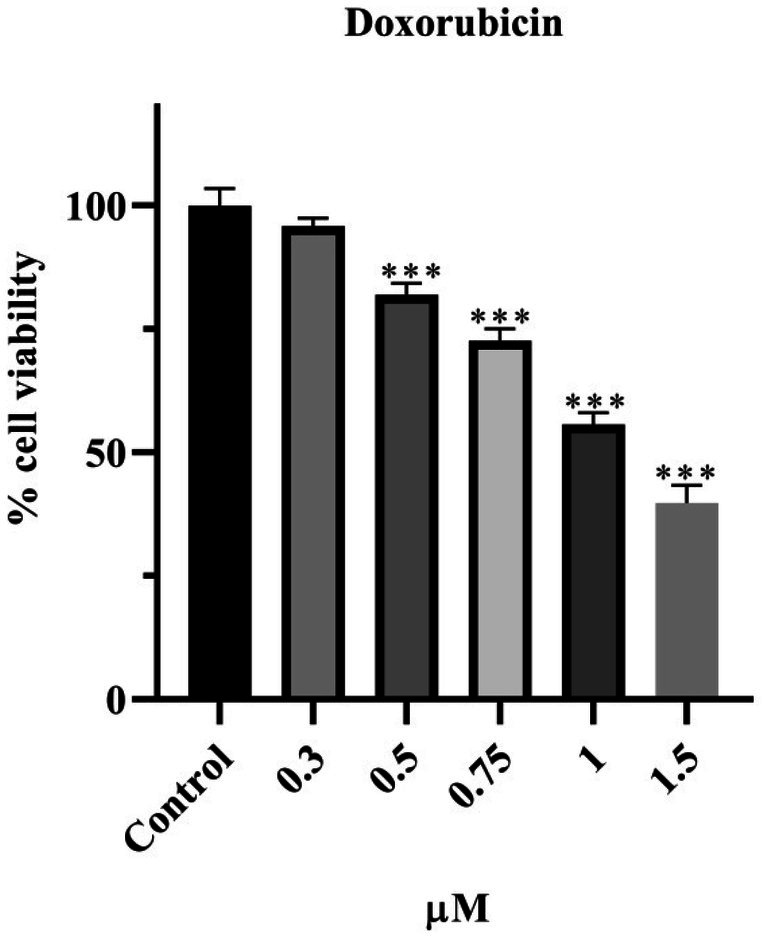
The 48 h effects of Doxorubicin on HeLa cell viability. Analyzed using One‐way ANOVA and post hoc Dunnett test in GraphPad Prism 8.3.0 program. Groups are given as fold change compared to control. *p* value < 0.05. ***< 0.001 (*n* = 3). Results are presented as mean ± standard error of the mean.

The effect of Dox on the viability of HeLa cells is presented in Figure 2 according to SRB results. Dox was applied at concentrations of 0.3, 0.5, 0.75, 1, and 1.5 μM for 48 h. It was observed that a concentration of 0.3 μM did not affect cell viability, whereas Dox reduced viability to below 80% at 0.5 (*p* < 0.001), 0.75 (*p* < 0.001), 1 (*p* < 0.001), and 1.5 μM (*p* < 0.001). The decrease in cell viability is inversely proportional to the increase in concentration. The IC_50_ value was calculated as 1.01 μM for 48 h.

**FIGURE 3:**
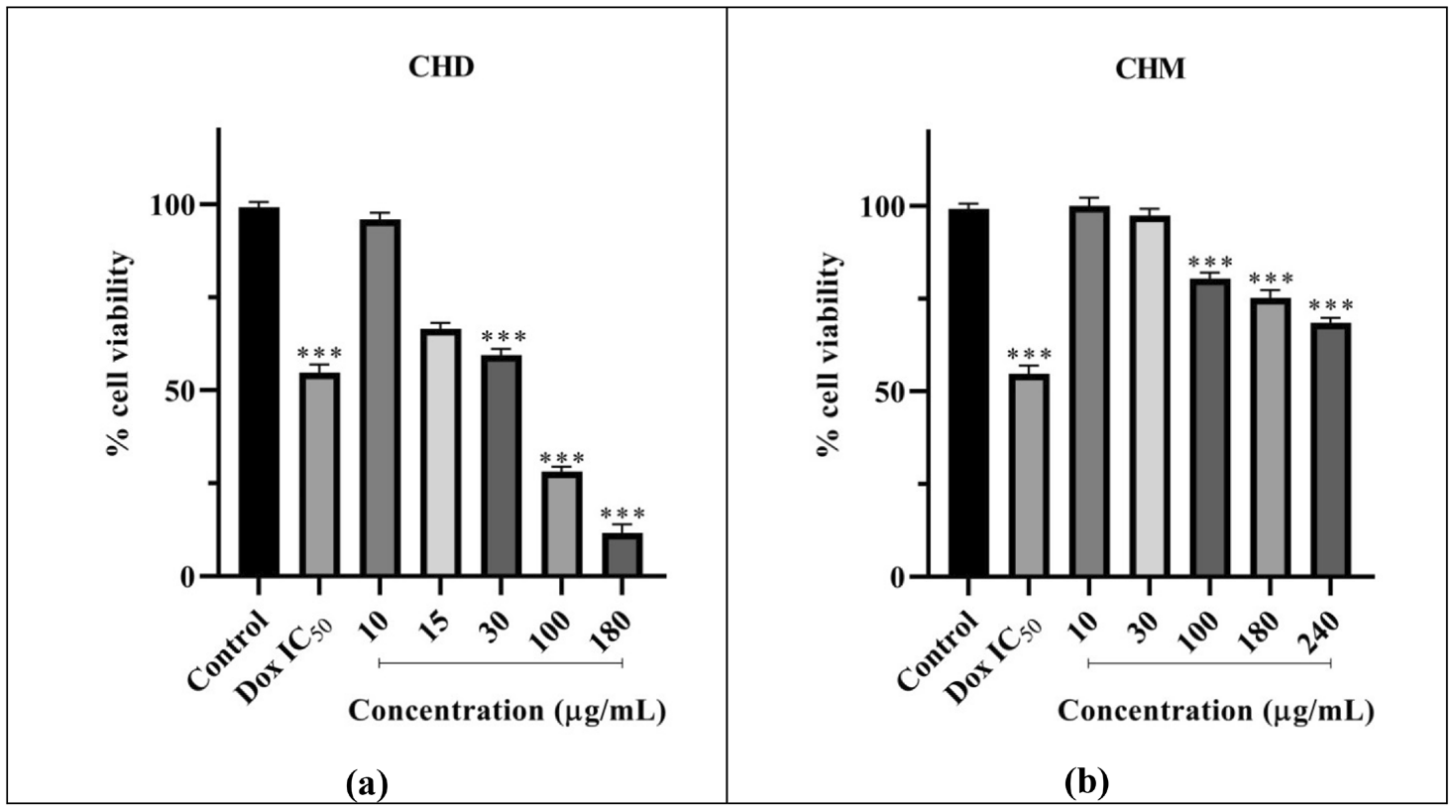
The 48 h effects of CHD (a) and CHM (b) on HeLa cell viability. Analyzed using one‐way ANOVA and post hoc Dunnett test in GraphPad Prism 8.3.0 program. Groups are given as fold change compared to control. *p* value < 0.05. Significance interval; ***< 0.001 (*n* = 3). Results are presented as mean ± standard error of the mean. CHD, Dichloromethane extract of *C. lycaonica*; CHM, Methanol extract of *C. lycaonica*; Dox IC_50_, Doxorubicin IC_50_ value.

The effect of CHD on the viability of HeLa cells is presented in Figure 3a. CHD was applied to HeLa cells at concentrations of 10, 15, 30, 100, and 180 μg/mL for 48 h, along with the Dox IC_50_ value previously determined. It was observed that CHD reduced viability to below 70% at 30 (*p* < 0.001), 100 (*p* < 0.001), and 180 μg/mL (*p* < 0.001), indicating cytotoxic effects at these concentrations. Within this concentration range, the decrease in cell viability is inversely proportional to the increase in concentration. The 10 μg/mL concentration did not cause a significant reduction in cell viability compared to the control, while at 15 μg/mL, it reduced viability to below 80%. According to SRB assay results, CHD's 48 h IC_50_ value was calculated as 39.27 μg/mL (Table 1).

**TABLE 1:** The IC_50_ values of Dox, CHD, and CHM were determined by SRB and xCELLigence RTCA system for 48 h.

Sample	RTCA IC_50_	SRB IC_50_
CHD	60.89 μg/mL	39.27 μg/mL
CHM	—	516.50 μg/mL
Dox	—	1.01 μM

The impact of CHM on the viability of HeLa cells is depicted in Figure 3b. CHM was administered at five different concentrations: 10, 30, 100, 180, and 240 μg/mL, along with the Dox IC_50_ for 48 h. It was observed that at 100 μg/mL (*p* < 0.001) and 180 μg/mL (*p* < 0.001), it reduced viability to below 80%, and at 240 μg/mL (*p* < 0.001), it decreased cell viability to 68.53%. A decrease in viability was observed with increasing concentration within this range. Viability did not significantly decrease compared to the control for 10 and 30 μg/mL. The IC_50_ value was calculated as 516.50 μg/mL for 48 h (Table 1).

### 3.2 Monitoring of Cytotoxicity of *C. lycaonica* Extracts in Real‐Time Using xCELLigence System

**FIGURE 4:**
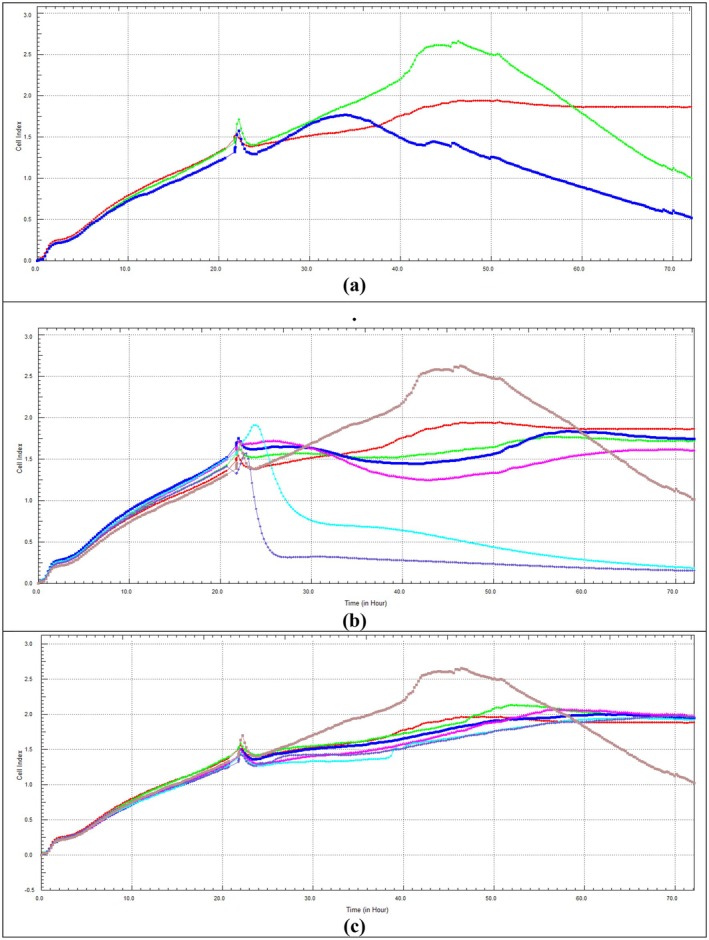
The concentration and time‐dependent effects of Dox, CHD, and CHM on HeLa cells using the xCELLigence RTCA system for 48 h: (a) Dox: Red: Control, Green: 1.00 μM, Blue: 1.50 μM. (b) CHD: Red: Control, Green: 10 μg/mL, Blue: 15 μg/mL, Pink: 30 μg/mL, Turquoise: 100 μg/mL, Purple: 180 μg/mL, Brown: Dox 1.00 μM. (c) CHM: Red: Control, Green: 10 μg/mL, Blue: 30 μg/mL, Pink: 100 μg/mL, Turquoise: 180 μg/mL, Purple: 240 μg/mL, Brown: Dox 1.00 μM. CHD: Dichloromethane extract of *C. lycaonica*, CHM: Methanol extract of *C. lycaonica*, Dox: Doxorubicin.

To evaluate Dox, CHD, and CHM effects on the changes in CI and the cell profiling in real time, the xCELLigence RTC experiment was performed (Figure 4) and calculated IC_50_ values (Table 1).

The effect of Dox on the viability of HeLa cells for 48 h using the xCELLigence RTCA system is given in Figure 4a. In the 48 h application, it was observed that Dox had a cytotoxic effect at concentrations of 1.00 and 1.50 μM and a decrease in viability was observed with the increase in concentration.

The effect of CHD on the viability of HeLa cells for 48 h using the xCELLigence RTCA system is given in Figure 4b. When comparing the 48 h application of CHD and the Dox IC_50_ with the SRB assay, it was observed that it reduced cell viability at 10 and 15 μg/mL and showed a cytotoxic effect at 30, 100, and 180 μg/mL concentrations. The decrease in cell viability was directly proportional to the increase in concentration. The 48 h IC_50_ value of CHD was calculated as 60.89 μg/mL (Table 1).

The effect of CHM on the viability of HeLa cells for 48 h using the xCELLigence RTCA system is given in Figure 4c. When CHM was applied for 48 h and compared with the Dox IC_50_ value at the SRB assay, the decrease in cell viability was directly proportional to the increase in concentration.

The 48 h IC_50_ values of CHD and CHM applications calculated with SRB and xCELLigence RTCA software are shown in Table 1. The data from the SRB analysis were consistent with the results obtained from the xCELLigence RTCA system.

### 3.3 Apoptotic Analysis of *C. lycaonica* Extracts

#### 3.3.1 Caspase‐3 Assay

**FIGURE 5:**
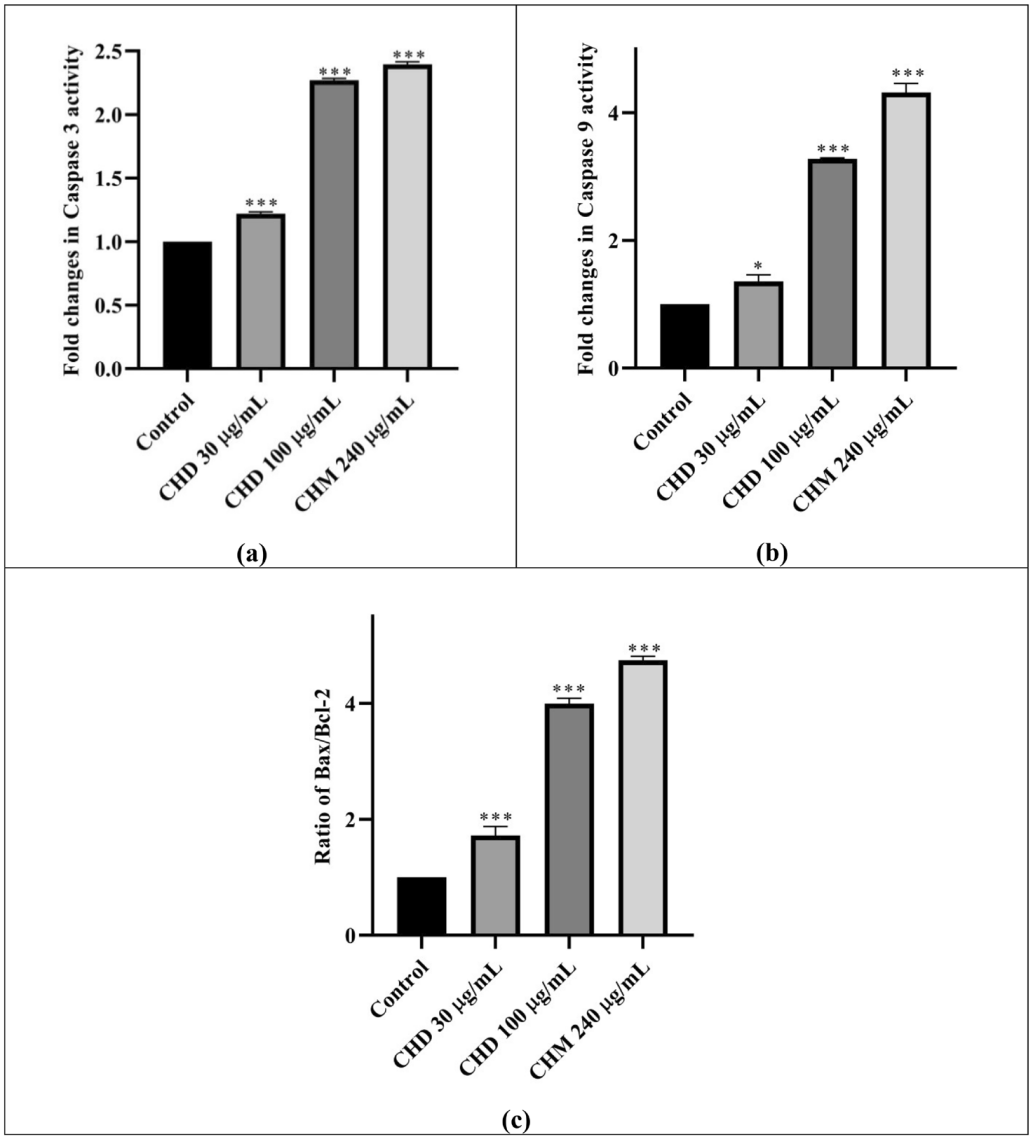
Fold changes of (a) Caspase‐3, (b) Caspase‐9 activity, and (c) Bax/Bcl‐2 ratio for CHD and CHM applications on HeLa cells. Analyzed using one‐way ANOVA and post hoc Dunnett test in GraphPad Prism 8.3.0 program. Groups are given as fold change compared to control. *p* value < 0.05. Significance interval; *** < 0.001 (*n* = 3). Results are presented as mean ± standard error of the mean. CHD: Dichloromethane extract of *C. lycaonica*, CHM: Methanol extract of *C. lycaonica*.

The change in caspase‐3 activity of CHD and CHM on HeLa compared to the control is given in Figure 5a and Table 2. It was observed that the caspase‐3 activity increased by 1.22 and 2.27 fold compared to the control at CHD 30 and 100 μg/mL concentrations, respectively; at the same time, it was found that the activity increased by 2.40 fold after 48 h of applying CHM 240 μg/mL (Table 2). Both extracts increased the caspase‐3 activity very highly statistically significantly (proportionally to the concentration) compared to the control.

**TABLE 2:** Fold change of Caspase‐3, Caspase‐9, and Bax/Bcl‐2 ratio on HeLa cells after CHD and CHM application.

Sample	Caspase‐3 fold changes	Caspase‐9 fold changes	Bax/Bcl‐2 ratio changes
CHD 30 μg/mL	+1.22	+1.36	+1.22
CHD 100 μg/mL	+2.27	+3.28	+3.46
CHM 240 μg/mL	+2.40	+4.32	+4.74

#### 3.3.2 Caspase‐9 Assay

The change in Caspase‐9 activity of CHD and CHM compared to the control is given in Figure 5b and Table 2. It was found that CHD 30 and 100 μg/mL doses increased Caspase‐9 activity by 1.36 and 3.28 times, respectively. The change in Caspase‐9 at CHM 240 μg/mL concentration was 4.28 fold. Although the increase in CHD was significant in itself, it increased in a concentration‐dependent manner (Table 2).

#### 3.3.3 Bax/Bcl‐2 Ratio

Bax and Bcl‐2 activities were measured using separate assays. The results then were proportioned to each other. The changes compared to the control are given in Figure 5c and Table 2. According to the data, a very highly statistically significant increase was observed in all results. It was seen that the Bax/Bcl‐2 ratio increased as the concentration increased in CHD. At CHD 30 and 100 μg/mL concentrations, the ratio increased by 1.72 and 3.46 times compared to the control, respectively. At CHM 240 μg/mL concentration, an increase of 4.74 times was observed (Table 2).

### 3.4 LC‐HRMS Results

**FIGURE 6:**
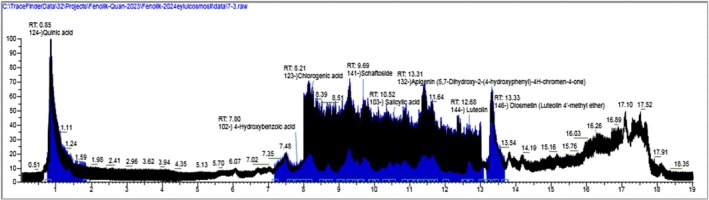
TIC profile of standard compounds and CHM extract. (The black chromatogram: The total ion chromatogram (TIC) of the standard compound mixture. The blue chromatogram: The total ion chromatogram (TIC) of the CHM extract).

**FIGURE 7:**
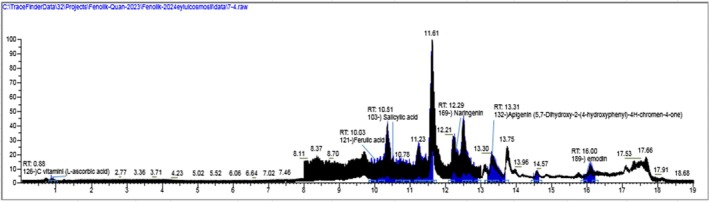
TIC profile of standard compounds and CHD extract. (The black chromatogram: The total ion chromatogram (TIC) of the standard compound mixture. The blue chromatogram: The total ion chromatogram (TIC) of the CHD extract).

The LC‐HRMS analysis performed to identify the compounds responsible for the activity in the extracts are given in (Table 3) and (Figures 6 and 7). According to the results, chlorogenic acid (3800.048 μg/g_extract_), quinic acid (3699.05 μg/g_extract_), 3‐(4‐hydroxyphenyl) propionic acid (2942.334 μg/g_extract_) and apigenin (1388.982 μg/g_extract_) were determined as a major compound in CHM extract and diosmetin (1573.298 μg/g_extract_) and apigenin (639.052 μg/g_extract_) in CHD extract. As well as diosmetin, apigenin, and 3‐(4‐hydroxyphenyl) propionic acid were the common compounds in both extracts. Although chlorogenic acid and quinic acid were found in the CHM extract as major compounds, they were not detected in the CHD extract.

**TABLE 3:** The LC‐HRMS results of CHM and CHD extracts.

Compound No	Compound name	RT (min)		[M^−^H]^−^ (*m/z*)	Content (μg/g_extract_)	
		CHM	CHD		CHM	CHD
1	4‐Hydroxybenzoic acid	7.8	N/F*	137.02442	46.826	0.965
2	Salicylic acid	10.52	10.51	137.02442	51.554	—
3	3‐Hydroxybenzoic acid	N/F	N/F	137.02442	—	—
4	3‐Hydroxyphenylacetic acid	N/F	N/F	107.05053	—	—
5	Syringic acid	8.9	N/F	197.04555	3.882	—
6	Gallic acid	N/F	N/F	169.01425	—	—
7	Protocatechuic acid	N/F	N/F	153.01933	—	—
8	Protocatechuic acid ethyl ester	N/F	N/F	181.05063	—	—
9	3,4‐Dihydroxybenzaldehyde	N/F	N/F	137.02442	—	—
10	2,4‐Dihydroxybenzoic acid	8.4	N/F	153.01933	105.114	—
11	Vanillic acid	8.52	N/F	167.03498	67.048	—
12	Homovanillic acid	N/F	N/F	181.05063	—	—
13	Vanillin	N/F	N/F	151.04007	—	—
14	Gentisic acid	N/F	N/F	153.01933	—	—
15	Homoprotocatechuic acid	N/F	N/F	167.03498	—	—
16	Trans Cinnamic acid	N/F	N/F	147.04515	—	—
17	Coumaric acid	N/F	N/F	163.04007	—	—
18	Caffeic acid	N/F	N/F	179.03498	—	—
19	Caffeic acid phenhyl ester	N/F	N/F	283.09758	—	—
20	Ferulic acid	10.03	10.03	193.05063	13.186	3.022
21	Sinapic acid	N/F	N/F	223.06120	—	—
22	Chlorogenic acid	8.21	N/F	353.08781	3800.048	—
23	Quinic acid	0.85	N/F	191.05611	3699.05	—
24	3‐(4‐Hydroxyphenyl) propionic acid	9.36	N/F	165.05572	2942.334	314.272
25	α‐Cyano‐4‐hydroxycinnamic acid	N/F	N/F	188.03532	—	—
26	Catechin	N/F	N/F	289.07176	—	—
27	Epigallocatechin	N/F	N/F	305.06668	—	—
28	Epigallocatechin gallate	N/F	N/F	457.07763	—	—
29	Chrysin	N/F	N/F	253.05063	—	—
30	Apigenin	13.31	13.31	269.04555	1388.982	639.052
31	Acacetin	N/F	N/F	283.06120	—	—
32	Rhoifolin	N/F	N/F	431.09891	—	—
33	Vicenin 2	9.24	N/F	593.15119	30.712	—
34	Apigenin 7‐glucuronide	11.38	N/F	445.07763	17818.7	0.358
35	Apigenin 7‐glucoside	N/F	N/F	431.09837	—	—
36	Genkwanin	N/F	N/F	283.06120	—	—
37	Apiin (Apigenin‐7‐(2‐O‐apiosylglucoside))	N/F	N/F	563.14063	—	—
38	Schaftoside	9.69	N/F	563.14063	28.866	—
39	Rutin	N/F	N/F	609.14611	—	—
40	Luteolin	12.68	12.69	285.04046	222.374	4.192
41	Luteolin‐7‐O‐glucuronide	N/F	10.86	461.07255	—	19.492
42	Diosmetin	13.33	13.31	299.05611	436.962	1573.298
43	Orientin	10.03	N/F	447.09328	12.882	—
44	Isoorientin	N/F	N/F	447.09328	—	—
45	Luteoloside	10.69	N/F	447.09328	132.476	—
46	Luteolin 7‐rutinoside	N/F	N/F	593.15119	—	—
47	Galangin	N/F	N/F	269.04555	—	—
48	Quercetin	N/F	N/F	301.03538	—	—
49	Isoquercitrin	10.96	N/F	463.08820	23.97	—
50	Narcissin	N/F	N/F	623.16176	—	—
51	Quercetin 3‐rutinoside 7‐glucoside	N/F	N/F	447.09296	—	—
52	Isorhamnetin	13.29	N/F	315.05103	10.404	—
53	Kaempferol	N/F	N/F	285.04046	—	—
54	Afzelin	12.15	N/F	431.09837	34.812	—
55	Kaempferide	N/F	N/F	299.05611	—	—
56	Nicotiflorin	N/F	N/F	593.15119	—	—
57	Astragalin	N/F	N/F	447.09328	—	—
58	Myricetin	N/F	N/F	317.03029	—	—
59	Fisetin hydrate	N/F	N/F	285.04046	—	—
60	Naringenin	N/F	12.29	271.06120	—	3.102
61	Sakuranetin	N/F	N/F	285.07685	—	—
62	Narirutin	N/F	N/F	579.17193	—	—
63	Metaflumizone	N/F	N/F	505.11020	—	—
64	Oleuropein	N/F	N/F	539.17633	—	—
65	Hesperidin	N/F	N/F	301.07138	—	—
66	Eriodictyol	N/F	N/F	287.05501	—	—
67	Eriocitrin	N/F	N/F	595.16684	—	—
68	Liquiritigenin	N/F	N/F	255.06628	—	—
69	Genistein	N/F	N/F	269.04555	—	—
70	Daidzin	N/F	N/F	415.10346	—	—
71	Formononetin (Neochanin)	N/F	N/F	267.06628	—	—
72	Ellagic acid	11.32	11.36	300.99899	16.05	11.546
73	Esculin hydrate	7.55	N/F	339.07216	16.35	—
74	Phloridzin	N/F	N/F	435.12967	—	—
75	Rosmarinic acid	N/F	N/F	359.07724	—	—
76	Glabridin	N/F	N/F	323.12888	—	—
77	Arbutin	N/F	N/F	271.08233	—	—
78	Emodin	N/F	N/F	269.04555	—	—
79	Pinocembrin	N/F	N/F	255.06694	—	—
80	Doxorubicin Hydrchloride	N/F	N/F	542.16678	—	—
81	Ethylgallate	N/F	—	197.04555	—	

* N/F: Not found.

**FIGURE 8:**
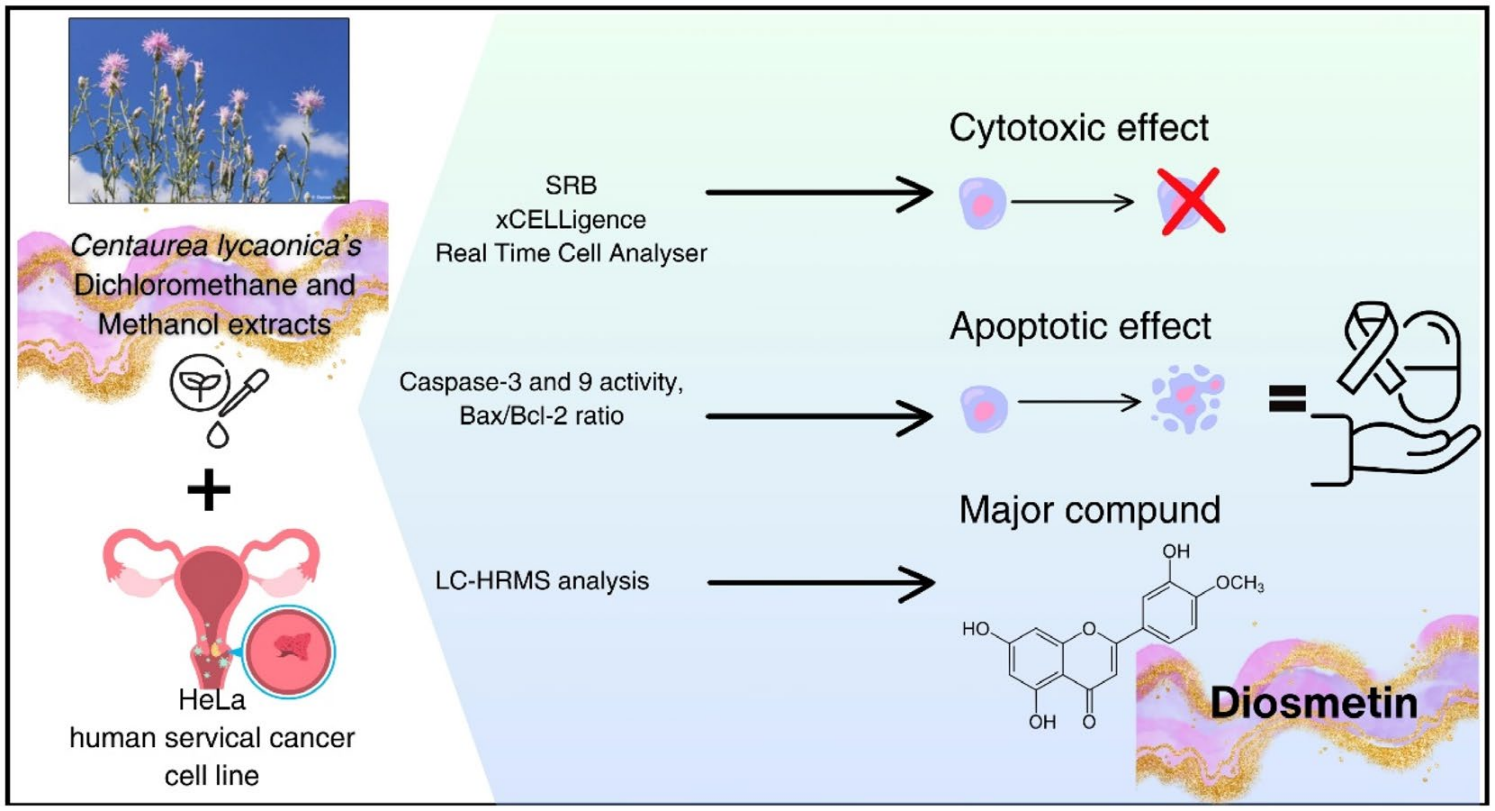
Apoptotic properties and related compounds of CHD and CHM.

## 4 Discussion

The Asteraceae family, commonly called the “sunflower family,” is among the largest groups of flowering plants. Numerous well‐known species of the Asteraceae family have been used in the human diet, such as sunflower, chicory, lettuce, and daisy (Teneva et al. 2024). Disease prevention through diet has been crucial in recent years; therefore, there is a growing demand for natural compounds derived from edible plants. Accordingly, there is an increasing interest in studying the Nutritional values of many members of the *Centaurea* genus as a part of the Asteraceae family to maximize its medicinal benefits and minimize its toxicity (Teneva et al. 2024). The *Centaurea* genus has been traditionally used for medicinal purposes (Honda et al. 1996; Sezik et al. 2001). It is known to have many different properties, such as anti‐inflammatory (Al‐Saghir et al. 2009; Erel et al. 2011; Garbacki et al. 1999; Koca et al. 2009; Negrete et al. 1984), antipyretic (Akbar et al. 1995), antimalarial (Sathiyamoorthy et al. 1999), antiviral (Bakr and Ayoub 2016), immunological (Arif et al. 2004) and vasodilatory effects (Orallo et al. 1998). Recent studies have indicated that various species within the *Centaurea* genus could show promising potential in cancer research (Artun and Karagöz 2021; Bakr and Ayoub 2016; Ceyhan Güvensen et al. 2019; Grafakou et al. 2022; Ozcan et al. 2016; Petropoulos et al. 2020; Sen et al. 2015; Yıldırım et al. 2022; Ahmed and Kamel 2014). While there are many studies to evaluate the cytotoxic effects of *Centaurea* genus extracts and their components on HeLa cells (Ahmed and Kamel 2014; Grafakou et al. 2022; Ozcan et al. 2016; Alper and Güneş 2019; Beltagy 2015; Bulut et al. 2021; Csapi et al. 2010; Csupor‐Löffler et al. 2009; Demirtas and Sahin 2013; Erenler et al. 2016; Erol‐Dayı et al. 2011; Kayacan et al. 2018; Kebbi et al. 2021; Köksal 2014; Uzun et al. 2017; Yaglioglu et al. 2014), there is no study in the literature yet to assess the cytotoxic and potential anticancer effect of *C. lycaonica* species extracts and their components on HeLa cells. The study is based on this gap.

Apoptosis is the programmed death of cells as an integral part of the normal cell cycle. In healthy cells, the apoptosis process is naturally initiated when cells need to end their lives, for example, when they are damaged, aged, or infected. So this prevents uncontrolled cell growth and maintains the homeostasis of the organism. However, cancer cells often disable apoptosis, leading to excessive growth and spread. Inhibiting or reducing apoptosis in cancer cells can increase tumor growth and metastasis. Therefore, understanding and targeting the mechanisms of apoptosis in cancer cells constitutes an important area in cancer treatment (Kari et al. 2022). There are several different methods to measure apoptotic activity. However, in recent years, a technique has emerged that is considered one of the most common and reliable methods to measure apoptotic activity in cell lines: measurement of caspase activity. Caspases are essential molecular processors for initiating, progressing, and completing apoptosis. These enzymes ensure that apoptosis occurs by cleaving certain substrates within the cell. Therefore, caspase activity is considered a direct indicator of the apoptotic process in the cell (Kari et al. 2022).

With the activation of caspases, apoptosis‐specific changes occur within the cell. As a result of caspase activity, apoptotic cells begin to shrink and undergo plasma membrane changes that signal a macrophage response (Gökhan et al. 2020). Caspases (cysteine aspartyl‐specific proteases) are cysteine proteins that cut target proteins. Caspase protease activity is crucial for successful apoptosis and cuts various proteins. There are four initiator caspases (Caspase‐2, ‐8, ‐9, 10) and three executioner caspases (Caspase‐3, ‐6, ‐7). Executioner caspases cut target proteins, ultimately leading to cell death (Sahoo et al. 2023).

A study in the literature investigates dichloromethane extract on the HeLa cell line belonging to *Centaurea* species (Şanal 2016). The study examined the anticancer activities of 4 different extracts from the stem part of *C. virgata* on the HeLa cell line at four different concentrations (5, 25, 50, 100 μg/mL). As a result of the study, it was observed that the most effective extract for HeLa was the dichloromethane extract (Şanal 2016). There are also a few studies investigating its cytotoxicity on different cell lines belonging to *Centaurea* species (Yirtici et al. 2017). The cytotoxic effects of dichloromethane extract prepared from the aerial parts of the *C. fenzlii* plant on the breast cancer cell line (MCF‐7) were determined using the MTT test for 48 h. The IC_50_ value was 122.521 μg/mL (Yirtici et al. 2017).

In the literature, 10 studies were found investigating methanol extract on HeLa cell line belonging to *Centaurea* species (Artun and Karagöz 2021; Bakr and Ayoub 2016; Ceyhan Güvensen et al. 2019; Yıldırım et al. 2022; Csapi et al. 2010; Csupor‐Löffler et al. 2009; Erol‐Dayı et al. 2011; Kayacan et al. 2018; Uzun et al. 2017; Yaglioglu et al. 2014). Considering that methanol extract is responsible for the cytotoxic effect of *Centaurea* species, many studies have shown that it is responsible for the cytotoxic effect of *Centaurea* species. And that the dichloromethane extract was responsible for the anticancer effect on the HeLa cell line of *C. virgata* ; methanol and dichloromethane extracts prepared from *C. lycaonica* were selected in our study, and it was revealed that dichloromethane was more effective than methanol extract and its cytotoxicity was due to its effect on apoptotic pathways.

SRB is a widely used method to determine cell viability. This method is designed to be highly sensitive to evaluate how cells respond to various conditions (Orellana and Kasinski 2016). The study preferred the SRB assay because it is simple, sensitive, inexpensive, and provides a better signal‐to‐noise ratio than formazan‐based assays (Keepers et al. 1991). Two studies in the literature use the SRB method to determine the effects of *Centaurea* on HeLa cancer cells (Beltagy 2015; Kebbi et al. 2021). The SRB assay determined the cytotoxicity of crude ethanol (95%) extracts of the aerial parts of the *C. scoparea* Sieb species on the HeLa cell line. The IC_50_ value was 27.5 ± 2.77 μg/mL for 24 h exposure (Beltagy 2015). In this study, the IC_50_ value of CHD was calculated as 39.27 μg/mL, and the IC_50_ value of CHM was calculated as 516.50 μg/mL using the SRB viability assay on HeLa cells.

The xCELLigence RTCA system enables continuous monitoring of dynamic processes such as cell adhesion, proliferation, and invasion using electrode‐based sensors (Kho et al. 2015). Only three studies used the xCELLigence RTCA system to evaluate the biological effects of *Centaurea* species on HeLa cells (Artun and Karagöz 2021; Bulut et al. 2021; Kayacan et al. 2018). One of the studies determined the cytotoxic effect of 95% methanol extract of the aerial part of *C. nerimaniae* species on the HeLa cell line using the xCELLigence RTCA system. The IC_50_ value after 48 h of extract exposure was determined to be 1.42 mg/mL (Kayacan et al. 2018). When the results of our study and the study conducted by Kayacan et al. (2018) with the *C. nerimaniae* species were evaluated together, it was seen that the plant part and the same methods were used; however, in our study, the *C. lycoanica* species had a much lower IC_50_ value that 60.89 μg/mL. However, it is thought that there may be differences depending on the plant species and the solvent used in obtaining the extract.

In another study, the potential cytotoxic effect of 95% methanol extract of the aerial part of *C. hermannii* on the HeLa cell line was investigated using xCELLigence RTCA, and the IC_50_ value for 48 h was found to be 18.3 μg/mL (Artun and Karagöz 2021). In our study, when compared with the results of the *C. hermanni* 's study, the IC_50_ value for the *C. lycoanica* species was not calculated for 48 h using the same method because it was higher than the used concentrations. It was concluded that the reason for the difference in the results may be different depending on the extract‐obtaining method and cell passage.

The potential cytotoxic effects of methanol, chloroform, and methanol‐chloroform extracts of leaves and seeds of *C. derderiifolia* were investigated in a study using the xCELLigence RTCA system, and it was revealed that the highest effect was in the chloroform extract of the leaf part with IC_50_ < 50 μg/mL (Bulut et al. 2021). In our study, the effect on the viability of HeLa cells using the xCELLigence RTCA system was calculated as 60.89 μg/mL for the dichloromethane extract, while the IC_50_ value of the methanol extract was not calculated because it was higher than the used concentrations. It is thought that the aerial part of *C. lycoanica* investigated in our study may have components that will show potential cytotoxic effects. The difference in the solvents used to prepare the plant extracts could have caused the different results.

In our study, the effects of CHD and CHM on apoptosis were evaluated by protein determination of Caspase‐9, the initiator caspase in the intrinsic pathway, and Caspase‐3, one of the caspases responsible for cutting cellular substrates. The most common method of measuring caspase activity is to determine the activity of caspase enzymes using ELISA kits. This method provides high sensitivity, accuracy, and reproducibility for cell measurements (Gökhan et al. 2020). Therefore, commercial kits based on ELISA measurement methods were preferred in our study. As a result, an increase was observed in the levels of both CHD and CHM when compared with the control group. In a study, the apoptotic effect of methanol extract (1.42 mg/mL) from the aerial parts of *C. nerimaniae* on HeLa cells was examined using the immunohistochemistry staining method, and Caspase‐3 activation was found to be significantly increased (Kayacan et al. 2018). In another study, to evaluate the apoptotic activity of 95% methanol extract of *C. hermannii* 's aerial part, two different concentrations were applied to HeLa cells (100 μg/mL) and 48 h IC_50_ value for HeLa cells (15.74 μg/mL). The effect of the extract on the activation of Caspase‐3, 7, and 9 was determined by the spectrofluorometric Caspase activity method. In the results obtained, the increase in Caspase‐9, 3, and 7 reveals that *C. hermannii* extract induced apoptosis in HeLa cells via the intrinsic pathway, and the results obtained showed the presence of apoptotic cell death (Artun and Karagöz 2021). In our study, the plant part used is the same as in the study conducted by Artun and Karagöz (2021), but the plant species and solvents used in obtaining the extract are different. However, the increase in caspase‐3 and 9 activities is similar. So this suggests that the aerial part of the *Centaurea* genus may have a cytotoxic effect by affecting the mechanisms associated with apoptosis.

Anti‐apoptotic Bcl‐2 proteins prevent apoptosis by inhibiting pro‐apoptotic Bcl‐2 proteins, namely Bcl‐2‐related X protein (Bax) and Bcl‐2 homologous antagonist killer (Bak) proteins (Zaman et al. 2014). Overexpression of Bcl‐2 protein is found in more than half of all cancers, regardless of their type. So this makes tumor cells resistant to any intrinsic apoptotic stimuli, including some anticancer drugs (Pfeffer and Singh 2018). In general, when an anticancer agent could induce apoptosis in cancer cells, a decrease in the levels of anti‐apoptotic Bcl‐2 proteins and an increase in the levels of pro‐apoptotic protein (Bax) are observed (Skommer et al. 2010). Our study evaluated the effects of CHD and CHM on apoptosis to determine Bax and Bcl‐2 proteins. As a result, while Bax protein levels increased, Bcl‐2 protein levels decreased, so an increase in the Bax/Bcl‐2 ratio was observed.

In the literature, no study investigates the apoptotic effects of dichloromethane extract of any *Centaurea* species on HeLa. However, there is research on different cell lines (Yirtici et al. 2017). The apoptotic and necrotic effects of the dichloromethane extract prepared from the aerial parts of *C. fenzlii* (IC_50_ value of 122.521 μg/mL) on MCF‐7 were evaluated under fluorescence microscopy with double staining. At the end of 48 h of application, it was observed that MCF‐7 cells generally went into early apoptosis (Yirtici et al. 2017). When the results of our study and the study revealed by Yirtici et al. (2017) were evaluated together, it was seen that the plant part and the same methods were used, and the *C. lycoanica* species had a lower IC_50_ value of 39.27 μg/mL. However, it is thought that there may be differences due to the difference in plant species and cell lines.

Doxorubicin is a powerful drug classified as an antineoplastic agent. It works by inhibiting the growth and proliferation of cancer cells. This provides a therapeutic effect by stopping or slowing down the uncontrolled growth of cancerous tissue. The use of doxorubicin in cancer research has become an important tool in the fight against many different types of cancer. It is effective in cancer types such as breast cancer, leukemia, lymphoma, and soft tissue sarcoma. This drug is used both alone and as part of treatment regimens in combination with other cancer drugs (Rivankar 2014). It has also been used as a positive control by researchers in anticancer research due to its confirmed effectiveness at low doses (Pandey et al. 2010; Wu et al. 2021; Zhang et al. 2021). Our study performed a general concentration screening using the SRB test. The results were found to be similar to the literature (Zhang et al. 2021). Dox was used as a positive control at a concentration of 1 μM in the SRB assay and the xCELLigence RTCA experiments.

Surgery, radiotherapy, chemotherapy, and combinations of these methods are used in the treatment of cervical cancer, which is one of the most common gynecological cancers in women. The inadequacy of current treatments and the low tolerance of chemotherapy by patients reveal the necessity of new treatment methods. For this purpose, in the study carried out by us, the cytotoxic and apoptotic mechanisms of action of dichloromethane and methanol extracts obtained from the *C. lycaonica* plant on the HeLa cell line were investigated, and the potential anticancer activity data of the plant were revealed for the first time. CHD showed a cytotoxic effect at concentrations of 30; 100, and 180 μg/mL, while CHM showed a cytotoxic effect at 240 μg/mL at 48 h. The concentrations of CHD 10; 15 μg/mL and CHM 10; 30 μg/mL didn't show cytotoxic effect. According to the results of the SRB assay, IC_50_ values were calculated as 39.27 μg/mL for CHD and 516.50 μg/mL for CHM; and according to xCELLigence RTCA results, IC_50_ value is 60.89 μg/mL for CHD. This difference in IC_50_ values arises from the different principles of the methods by which cell viability is measured. The SRB assay, as an end‐point test (Vichai and Kirtikara 2006), is a method that obtains spectroscopic data by treating cells with dye and organic solvents, which is used to measure cell viability. The xCELLigence system is a method in which the entire process of changes in cell viability is followed electrochemically and in real time; no dye is used, and new and more data can be obtained (Lamarche et al. 2020). In addition to the precise detection of the onset time of cell death and early changes in morphology with the xCELLigence RTCA system, it has the advantage that the measurement can be made without damaging the cells (Atmaca et al. 2016). In this study, the real‐time profile of the cytotoxic effect of CHD and CHM on HeLa cells was created in the xCELLigence RTCA system; it was observed that an apoptotic effect was induced in HeLa cells treated with CHD, depending on the increasing concentration. When IC_50_ values were compared with previous studies on *Centaurea* species (Artun and Karagöz 2021; Beltagy 2015; Bulut et al. 2021; Kayacan et al. 2018; Kebbi et al. 2021; Yirtici et al. 2017), it was shown for the first time that the concentrations of CHD and CHM stated to have cytotoxic effects (30 and 100 μg/mL for CHD; 240 μg/mL for CHM) and significantly induced apoptotic cell death with an increase in Caspase‐3, 9 activity, and Bax/Bcl‐2 ratio, and it was thought that the reason behind this cytotoxic effect is a specific compound identified using the LC‐HRMS analysis. The major compounds found in CHM were chlorogenic acid, quinic acid, 3‐(4‐hydroxyphenyl) propionic acid, and apigenin. However, the major compounds found in CHD were diosmetin and apigenin. The biological activities of plant extracts, including their pro‐apoptotic and cytotoxic effects, are largely influenced by the chemical nature and concentration of their secondary metabolites (Özay and Pehlivan 2024).

Numerous phytochemical investigations on different *Centaurea* species have revealed a wide spectrum of secondary metabolites such as sesquiterpene lactones, flavonoids, lignans, phenolic acids, and alkaloids. In a study, lignans such as arctiin and matairesinol, flavonoids like astragalin and afzelin, and novel indole alkaloids like schischkiniin. These species exhibited notable cytotoxicity and antioxidant activity, often attributed to such metabolite diversity (Shoeb 2005). In another study, methanol extract and ethyl acetate fraction of *C. lycaonica* revealed the presence of bioactive compounds such as apigenin, myristoleic acid, malvidin 3‐galactoside, phloretin 2′‐xyloglucoside, and caffeic acid derivatives (Fatullayev et al. 2023). Although, dried and powdered aerial parts of *C. pannonica* were extracted by cold maceration using a solvent mixture of cyclohexane, diethyl ether, and methanol (1:1:1, v/v/v). The crude extract was subsequently partitioned with brine (saturated aqueous NaCl solution). The obtained lipophilic extract was subjected to a series of chromatographic procedures and along with the guainolides: babylin A, chlorohyssopifolin C janerin, 19‐deoxyjanerin, the flavonoids also obtained such as apigenin, diosmetin, hispidulin, nepetin (Milošević Ifantis et al. 2013). Thus, the novelty of the present study lies in the identification of diosmetin, which was not previously reported in studies on *C. lycaonica*. These phytochemical profiles can vary substantially depending on environmental and geographical variables such as climate, altitude, soil characteristics, and growth stage of the plant. Furthermore, the prior isolation of this compound from *C. pannonica* supports the findings of the current study and is consistent with existing literature data.

Natural compounds exhibit a broad range of anticancer effects, including inducing apoptosis and autophagy and inhibiting the proliferation of cancer cells (Chirumbolo et al. 2018; Patel et al. 2013). Diosmetin (3′, 5, 7‐trihydroxy‐4′‐methoxyflavone) is an aglycone of the flavonoid glycoside diosmin widely distributed in natural plants (Raza et al. 2024). It is found mainly in citrus fruits, olive leaves, and extracts of many medicinal herbs (Chen et al. 2019). Diosmetin has been shown to induce apoptosis in various types of cancer, including colon, liver, breast, leukemia, lung, prostate, and skin cancers (Choi et al. 2019; Koosha et al. 2019; Ma and Zhang 2020; Oak et al. 2018; Roma et al. 2018; Wang et al. 2019; Liu et al. 2016). Studies have reported that diosmetin decreases Bcl‐2 expression while increasing Bax and Bak expression in liver, lung, breast, gliomas, and colon cancers (Ma and Zhang 2020; Liu et al. 2016; Qiao et al. 2016). Additionally, another study found that diosmetin induces apoptosis in colon cancer cells by activating caspase‐3 and caspase‐9 (Koosha et al. 2019).

Among all studies done to investigate the metabolite composition of *Centaurea* species, only 4 studies for 4 different species (*C. vitrgata* Lam., *C. paphlagonica* (Bornm.) Wagenitz, *C. pannonica* (Heuff.) Simonk. and *C. hyalolepis*) conducted the content of diosmetin. These studies employed various analytical techniques, including column chromatography, one‐dimensional and two‐dimensional Nuclear Magnetic Resonance (1D & 2D NMR), Electrospray Ionization Mass Spectrometry (ESI‐MS) and Liquid chromatography‐Orbitrap‐high‐resolution mass spectrometry (LC‐Orbitrap‐HRMS) (Bozkurt 1997; Tüfekçi et al. 2024; Milošević Ifantis et al. 2013; Ercan et al. 2025). In this study, LC‐HRMS analysis revealed that CHD and CHM extracts contain diosmetin, with CHD containing 1573.298 μg/g_extract_ and CHM containing 436.962 μg/g_extract_. Diosmetin is believed to be responsible for the cytotoxic effects observed in both extracts. The higher cytotoxic effect exhibited by CHD compared to CHM is likely due to its higher diosmetin content. As a result, since the increase in caspase‐3 and 9 activities and the Bax/Bcl‐2 ratio is considered an apoptotic marker (Klimentova et al. 2021), it was concluded that CHD has significant importance in cervical cancer research as a cytotoxic and apoptotic effective extract with its low IC_50_ value. In addition, it is revealed that the methanol‐soluble parts of the above‐ground part of the plant also have cytotoxic effects. This study determined the cytotoxic and apoptotic effects of methanol and dichloromethane extracts prepared from *C. lycaonica* on HeLa for the first time. This study is also the first to investigate the changes in caspase‐9 protein activity and Bax/Bcl‐2 ratio regarding cervical cancer with the *Centaurea* genus (Figure 8). There is no study in the literature that we can compare the study conducted with this species.

*C. lycaonica* extract demonstrates strong potential for application across various industries, including food, dietary supplements, and pharmaceuticals. This extract could contribute significantly to the development of innovative products. Further in‐depth studies on the plant's potential medicinal uses are necessary. *C. lycaonica* is classified as Endangered (EN) by the International Union for Conservation of Nature (IUCN) (Ekim et al. 2000).

## 5 Conclusion

In conclusion, the present study demonstrated that CHD inhibits the growth of HeLa cells in vitro by inducing apoptosis through the mitochondrial apoptotic pathway. The higher cytotoxic effect exhibited by CHD compared to CHM is likely due to its higher diosmetin content. However, further studies are needed to understand the apoptosis induction mechanism fully. This study is believed to encourage further advanced research on *C*. *lycaonica* species, such as exploring its mechanism of action.

## Author Contributions

**Ayşe Kübra Karaboğa Arslan:** conceptualization (lead), formal analysis (lead), funding acquisition (lead), investigation (equal), methodology (equal), project administration (equal), supervision (lead), writing – original draft (equal), writing – review and editing (equal). **Safa Eminoğlu:** conceptualization (equal), formal analysis (equal), investigation (equal), methodology (equal), project administration (equal), writing – original draft (equal), writing – review and editing (equal). **Leyla Paşayeva:** conceptualization (equal), funding acquisition (equal), methodology (equal), project administration (equal), writing – original draft (equal), writing – review and editing (equal). **Nuh Mehmet Bozkurt:** methodology (equal). **Osman Tugay:** resources (equal).

## Ethics Statement

The authors have nothing to report.

## Conflicts of Interest

The authors declare no conflicts of interest.

## Data Availability

The authors have nothing to report.
